# Improving bovine disease detection through multilabel classification

**DOI:** 10.1038/s41598-025-18491-z

**Published:** 2025-10-06

**Authors:** Ghalib Nadeem, Muhammad Fahim Ul Haque, Hameeza Ahmed, Mansoor Ebrahim, Sadique Ahmad, Isabel de la Torre Díez, Hanaa A. Abdallah, Abdelhamied A. Ateya

**Affiliations:** 1 Department of Electrical and Computer Engineering, Iqra University, Karachi, 75500 Pakistan; 2 Department of Telecommunications Engineering, NED University of Engineering and Technology, Karachi, 75270 Pakistan; 3 Department of Computer and Information Systems Engineering, NED University of Engineering and Technology, Karachi, 75270 Pakistan; 4 Faculty of Engineering Sciences and Technology, Iqra University, Karachi, 75500 Pakistan; 5 EIAS Data Science Lab, College of Computer and Information Sciences, Prince Sultan University, Riyadh, 11586 Saudi Arabia; 6 Department of Signal Theory and Communications, University of Valladolid, Valladolid, Spain; 7 Department of Information Technology, College of Computer and Information Sciences, Princess Nourah bint Abdulrahman University, P.O. Box 84428, Riyadh, 11671 Saudi Arabia

**Keywords:** Behavioral data analysis, Bovine disease detection, Classifier chains model, SMOTE, Computational biology and bioinformatics, Health care, Engineering, Mathematics and computing

## Abstract

**R1.C1**: The dairy industry is a cornerstone of global food production and economic development; yet, its productivity is frequently hindered by common bovine health issues, including lameness, mastitis, metritis, and foot-and-mouth disease. These conditions not only affect milk yield but also pose significant challenges to maintaining animal welfare, highlighting the urgent need for intelligent, data-driven monitoring systems. **R1.C2**: In response to this critical need, this research proposes a machine learning (ML)-based framework for the early detection of such bovine events and diseases through multi-label classification. **R1.C3**: The system identifies estrus, calving, lameness, mastitis, and acidosis by analyzing key behavioral metrics derived from sensor-based monitoring, including feeding duration, resting periods, locomotion patterns, and aggregated activity data. **R1.C4**: In the context of multi-label bovine disease prediction, the combination of SMOTE and Classifier Chains is particularly crucial and synergistic due to the nature of the data and the interdependent relationships among the labels. **R1.C5**: The system was tested using a large dataset of 2.35 million records of livestock behavioral metrics. **R1.C6**: Among the six machine learning models investigated, the classifier chain configuration utilizing an Extra Tree Classifier consistently demonstrated superior performance, achieving a remarkable 97% subset accuracy, 96% recall, 95% precision, 96% F1-score, and a minimal Hamming loss of 0.04. Therefore, it is evident that classifier chains combined with oversampling techniques can capture label correlations and improve prediction performance compared to standard binary relevance approaches.

## Introduction

According to the United Nations, the global population is projected to reach 9.7 billion by 2050, driving a surge in demand for livestock products. Currently, animal agriculture contributes 40% to global gross domestic product (GDP), and this growing dependence on animal products like meat, milk, and eggs underscores the need to maximize production efficiency^1^. The dairy and livestock sector plays a vital role in global income and nutrition, with milk production being a key commodity. However, milk production is significantly influenced by factors such as nutrition, management practices, lactation age, and environmental conditions. These include the season and year of calving, as well as genetic and environmental variables like climate and feeding. **R2.C6**: Additionally, performance depends on the stage of lactation, age, parity, breed, live weight, and milking frequency^2^. Timely management of these factors offers immense potential to enhance milk yield globally^3^.

Mastitis, lameness, calving, estrus, and acidosis are common afflictions in cattle, significantly affecting production within the dairy industry. Such conditions not only raise concerns about animal welfare but also lead to considerable economic losses due to reduced milk production, challenges in reproduction, and increased veterinary expenses^4^. A proper diagnosis system is essential for improving animal welfare, minimizing losses, and halting the spread of the disease. However, traditional diagnosis methods, such as manual inspection and veterinary inspections, often yield delayed or inaccurate results due to their reliance on symptoms that may not always be overt. Moreover, large herds are challenging to monitor many diseases and their variable frequency confounds the problem of diagnosis.

**R1.C12**: Although recent research has examined successful strategies like time-series models for calving prediction^5^ and camera-based lameness detection^6^, these approaches focus on isolated health events and may not be scalable across all farms. Recent advancements in machine learning (ML) can aid in identifying diseases by analyzing animal behavior, physiological data, and images^7^. Acidosis is much less common than estrus and mastitis, which can skew models towards more frequent conditions, diminishing their effectiveness in identifying rarer diseases. Moreover, the interdependence of labels means that conditions like lameness can heighten the risk of mastitis due to decreased mobility^8^. Overlooking these relationships can result in less accurate predictions. To tackle these issues, hybrid approaches such as the synthetic minority over-sampling technique (SMOTE) for data balancing and classifier chains for capturing label dependencies have proven effective^9,10^.

The purpose of this work is to design an ML framework that works based on behavioral data, allowing the identification of multiple diseases simultaneously in bovine farms. This system applies multi-label classification techniques to predict different diseases at the same time, making use of a combination of under-sampling and oversampling methods to handle the imbalanced datasets and classifier chains for dependence between labels. It is trained on four distinct datasets taken from bovine farms^11^. The paper proposes a pipeline in which single model classifier chains (SMCC) combine various classifiers, including random forest (RF), histogram gradient boosting classifier, extreme gradient boost, light gradient boost model, categorical boost classifier, and extra trees classifier. SMOTE intervened to address the problem of data imbalance in the training process of the model, which guarantees the representation of all health conditions in the model. Results are evaluated by multi-label classification metrics, namely Hamming loss, subset accuracy, macro-averaged precision, recall, and F1-score. One can see that the combination of SMOTE and SMCC enables better results. The extra tree was capable of detecting calving and estrus events with 99% accuracy, precision, and recall, 0.04 hamming loss, and a subset accuracy of 97%.

This proposed work is the first to present a full framework to detect bovine disease and health events using a multi-label single-shot classifier chain to compile diverse ML models with custom hyperparameter optimization. As the captured dependencies among health conditions such as calving, estrus, mastitis, acidosis, and lameness are identified, this framework can bypass the limitations posed by traditional binary or multi-class classification techniques and provide for a much more holistic and accurate prediction. Key challenges in livestock health monitoring, such as data imbalance, feature relevance, and predictive accuracy for rare events, are addressed through advanced strategies, including SMOTE for balancing class representation and an evaluation pipeline employing metrics like hamming loss, precision, recall, and subset accuracy. By providing a non-invasive, cost-effective, and stress-free alternative to traditional methods, this AI-based system simultaneously detects multiple diseases and events, allowing for timely interventions, reducing economic losses, improving bovine welfare, and preventing disease spread^12^. To the best of our knowledge, the proposed work is the first to detect bovine diseases using a multi-label single-shot classifier chain, capturing label interdependencies for improved prediction accuracy. The main contributions of this paper are listed as follows.

1. Detecting bovine diseases using a *multi-label single-shot classifier chain*, capturing label interdependencies for improved prediction accuracy.
2. Using SMOTE to effectively mitigate class imbalance, ensuring balanced learning across majority and minority labels.
3. Employing comprehensive tuning of key parameters for each model to balance bias-variance trade-offs in multi-label classification.
4. Utilizing behavioral metrics (e.g., EAT, REST, IN_ALLEYS) as inputs, demonstrating their efficacy in predicting critical bovine diseases.
5. Performs evaluation using hamming loss, subset accuracy, recall, and f1-score, ensuring a robust assessment of model performance.
6. Facilitating early detection of bovine diseases and health events.

## Related work

Several researchers have investigated ML approaches to predict the onset of numerous health disorders. J. Wang et al.^13^ used ML to detect the estrus in dairy cows. The performance comparison of different ML algorithms, including support vector machine (SVM), artificial neural networks (ANN), and back-propagation neural networks (BPNN), has been done using data from a herd of dairy cows. The results show that the BPNN algorithm with a time window of 0.5 h has the highest accuracy in estrus detection. Similarly, N. Wagner et al.^14^ used ML algorithms, including k-nearest neighbors for regression (k-NNR), decision trees for regression (DTR), multi-layer perceptron (MLP), and long short-term memory (LSTM), to identify anomalous behavior in dairy cows suffering from sub-acute ruminal acidosis (SARA).

X. Zhou et al.^15^ used extreme gradient boost (XGB), RF, k-NNR, and Rpart algorithms for the early prediction of lameness, mastitis, and metritis in dairy cows. In^16^, the authors employed RF, SVM, and NN algorithms to differentiate between diseased, reproductive, and stress situations in cows on sensor-based activity data. Another study in^14^ uses k-NNR and reports an accuracy of 83% for abnormal behavior detection, which is lower compared to other models. Studies by^10,17^ apply RTLS to predict bovine events. While these studies cover a broader range of events, the performance metrics remain partially unavailable, making direct comparisons difficult. The objectives of these studies vary significantly from detecting reproductive, pathological, and stress statuses^16^ to circadian rhythm changes^18^, abnormal behavior^14^, and broader psychological and reproductive event predictions^17,19^.

Table 1 provides a comprehensive summary of various studies on bovine events and disease prediction, highlighting per-label classifiers; however, Table 2 compares the considered performance metrics. However, many previous studies do not report these metrics comprehensively. Each study focuses on different aspects of bovine health, behavior, and event prediction, spanning multiple works published between 2020 and 2024. The study by^16] and [19^, using RF, achieved notable performance with 95.9% and 98.25% accuracy, respectively, alongside 97% precision, demonstrating the effectiveness of this model in discerning reproductive, pathological, and stress-related statuses. Similarly^20^, employed an FBAT method for circadian rhythm detection, achieving 90% accuracy.

Notably, the proposed method is favorable to these prior works. The combination of the classifier chain model (CCM) along with SMOTE used in the proposed approach demonstrates superior performance across almost all evaluation metrics, with an accuracy, recall, precision, and f1-score of 99%, 100%, 99%, and 100%, respectively. Additionally, the Hamming loss is 0.04, and the subset accuracy, macro recall, macro precision, and macro F1-score are 97%, 96%, 95%, and 96%, respectively. This advancement is particularly evident in the CCM’s ability to capture label dependencies, outperforming widely used models like RF and RTLS, which have been traditionally popular in bovine event prediction studies. The results confirm the significant contribution of the proposed method, establishing it as a benchmark for future work in bovine disease and event prediction. The superior performance of the CCM highlights its potential for providing accurate, real-time insights into bovine health, marking a substantial improvement over the previously published models.

**Table 1:** A summary of the comparison among different methods and techniques for event detection.

Ref.	Objective	Per-Label Classifier Performance				
		Models	Accuracy	Recall	Precision	F1-Score
^10^	The study links b/w behavior, health, and reproductive events.	RTLS	N/A	N/A	N/A	N/A
^14^	Detect abnormal behavior.	k-NNR	83%	N/A	N/A	N/A
^16^	Discernment b/w reproductive, pathological, and stress status.	RF	95.9%	95.4%	97%	N/A
^17^	Predict psychological and pathological events.	RTLS	N/A	N/A	N/A	N/A
^18^	Detect changes in circadian activity rhythm.	FBAT	90%	80%	N/A	N/A
^19^	Investigation of bovine events and diseases.	RF	98.25%	100%	97%	98%
Proposed	SMCC-based bovine event disease detection.	CCMs and SMOTE	99.02%	100%	99%	100%

**Table 2:** A summary of performance comparison among different methods and techniques.

Ref.	Objective	Overall Performance					
		Hamming Loss	Subset Accuracy	Macro Recall	Macro Precision	Macro F1-Score	Events
^10^	The study links b/w behavior, health, and reproductive events.	N/A	N/A	N/A	N/A	N/A	11
^14^	Detect abnormal behavior.	N/A	N/A	N/A	N/A	N/A	N/A
^16^	Discernment b/w reproductive, pathological, and stress status.	N/A	N/A	N/A	N/A	N/A	5
^17^	Predict psychological and pathological events.	N/A	N/A	N/A	N/A	N/A	11
^18^	Detect changes in circadian activity rhythm.	N/A	N/A	N/A	N/A	N/A	All
^19^	Investigation of bovine events and diseases.	N/A	N/A	N/A	N/A	N/A	5
Proposed	SMCC-based bovine events disease detection.	0.04	97%	96%	95%	96%	5

## Dataset description

The dataset used for this study is of dairy cows in barns collected from the CowView system (GEA Farm Technology, Bonen, Germany)^10^. The data comprises 386 distinct Holstein breed cattle in four separate datasets^10^. There exists a total of eleven events; the frequency of each event in a dataset is shown in Table 3. These events are divided into 3 states, linked to six health classes: lameness, mastitis, LPS, subacute ruminal acidosis, other diseases, and accidents. Estrus and calving are linked to reproduction, whereas stress events are animal mixing, disturbance, and marginal management changes. Among all the events, the suitable ones for the training, validating, and testing of ML models are calving, estrus, mastitis, and lameness; the remaining factors are for the farms observed during the data gathering^10^. Datasets 1 and 2, which included 28, 28 cows observed over 6 months and 2 months, were provided by the INRAE Herbipôle experimental farm in Marcenat, France, while datasets 3 and 4 were provided by commercial farms in Europe, 30, 300 cows observed over 40 days and 1 year. These 2,351,342 records cover a wide range of cow behaviors and health conditions, with behavioral features such as EAT (eating time), REST (resting time), ACTIVITY-LEVEL (General activity level), and IN-ALLEYS (movement in farm alleys). The target variables are multi-label binary classifications representing various health conditions, including estrus, calving, lameness, mastitis, and acidosis.

### Challenges with the dataset

Two major challenges in this dataset are imbalanced data and correlated labels, as diseases like acidosis occur much less frequently than others, making it difficult for the model to learn and recognize these rare conditions, leading to a significant class imbalance problem. This dataset imbalance could cause traditional ML models to perform poorly, especially for the minority classes. Similarly, diseases are often interdependent, as lameness can increase the risk of mastitis due to reduced mobility. These correlations complicate the classification task, as a simple independent model fails to capture such relationships.

**Table 3:** A summary of comparison among different methods and techniques for event detection.

Events	Occurrence			
	Dataset-1	Dataset-2	Dataset-3	Dataset-4
Calving	8	0	0	171
Accidental Events	0	0	0	15
Estrus	41	7	26	257
Lameness	4	16	0	114
Mastitis	9	3	0	32
LPS injection	27	0	0	0
Ruminal Acidosis	0	271	0	0
Mixing	72	0	0	0
Other Disturbance	173	671	0	12,223
Management Changes	0	168	0	2581
Other Disease	10	8	0	66

## Methodology

**Fig. 1:**
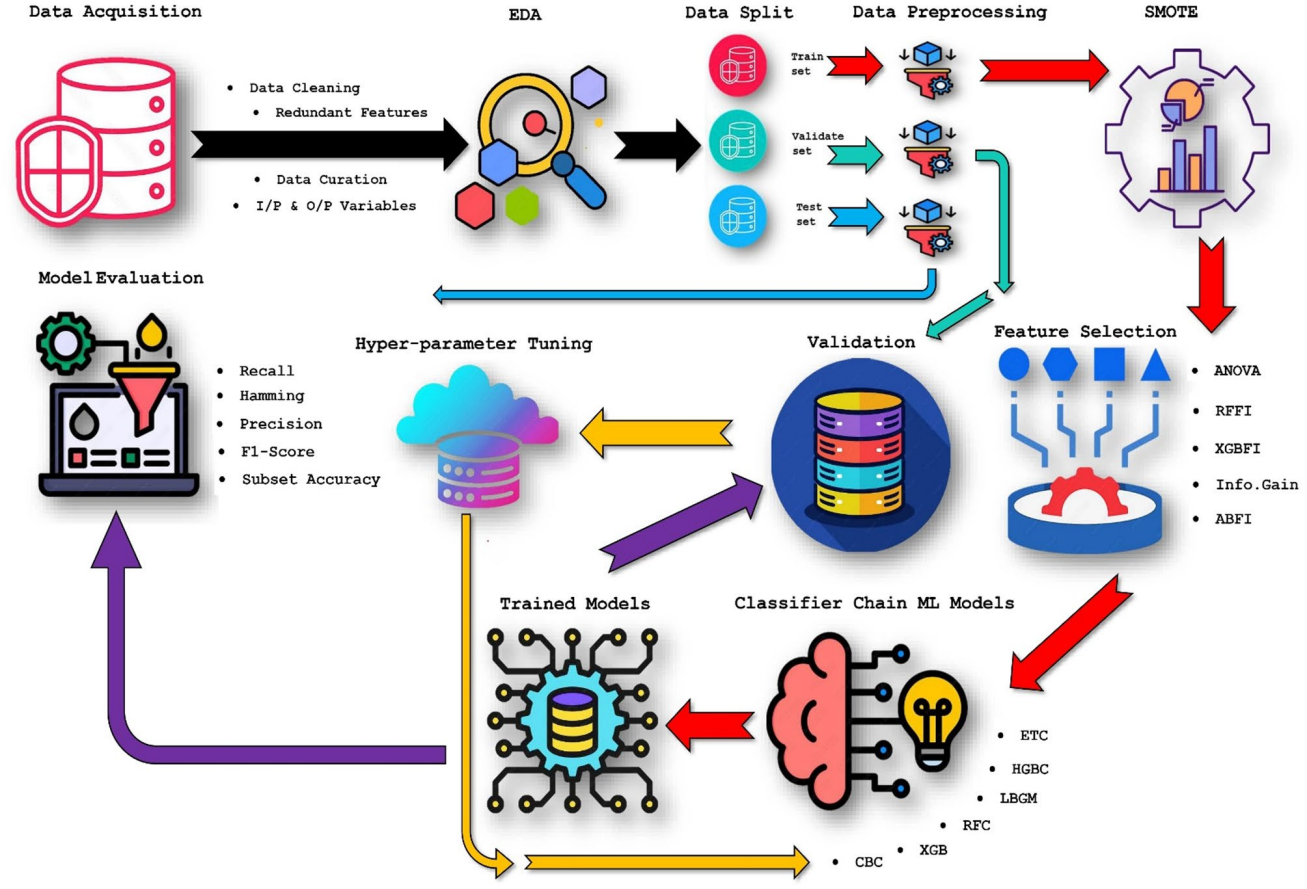
Workflow of the proposed ML-based pipeline of Bovine event and disease detection^30^.

The workflow includes data preprocessing, class imbalance handling, feature selection, model training, and evaluation. Orchestrating this entire process leverages state-of-the-art tools and methodologies to derive meaningful insights from intricate datasets. Figure 1 presents the overall flow of this pipeline.

### Data acquisition

This work collects raw data related to bovine behavior and health from^10^, as discussed in Sect. 3. The dataset includes four primary input features: EAT, REST, IN-ALLEYS, and ACTIVITY-LEVEL, which are indicators of bovine activity. The target output variables correspond to multiple bovine health conditions that we aim to predict: calving, estrus, mastitis, acidosis, and lameness. Before proceeding to modeling, the data undergoes an initial cleaning process. Redundant or irrelevant features are removed, and missing values are handled to ensure the data is suitable for further analysis. This step also involves basic data curation, clearly defining the input and output variables.

### Exploratory data analysis

**Fig. 2:**
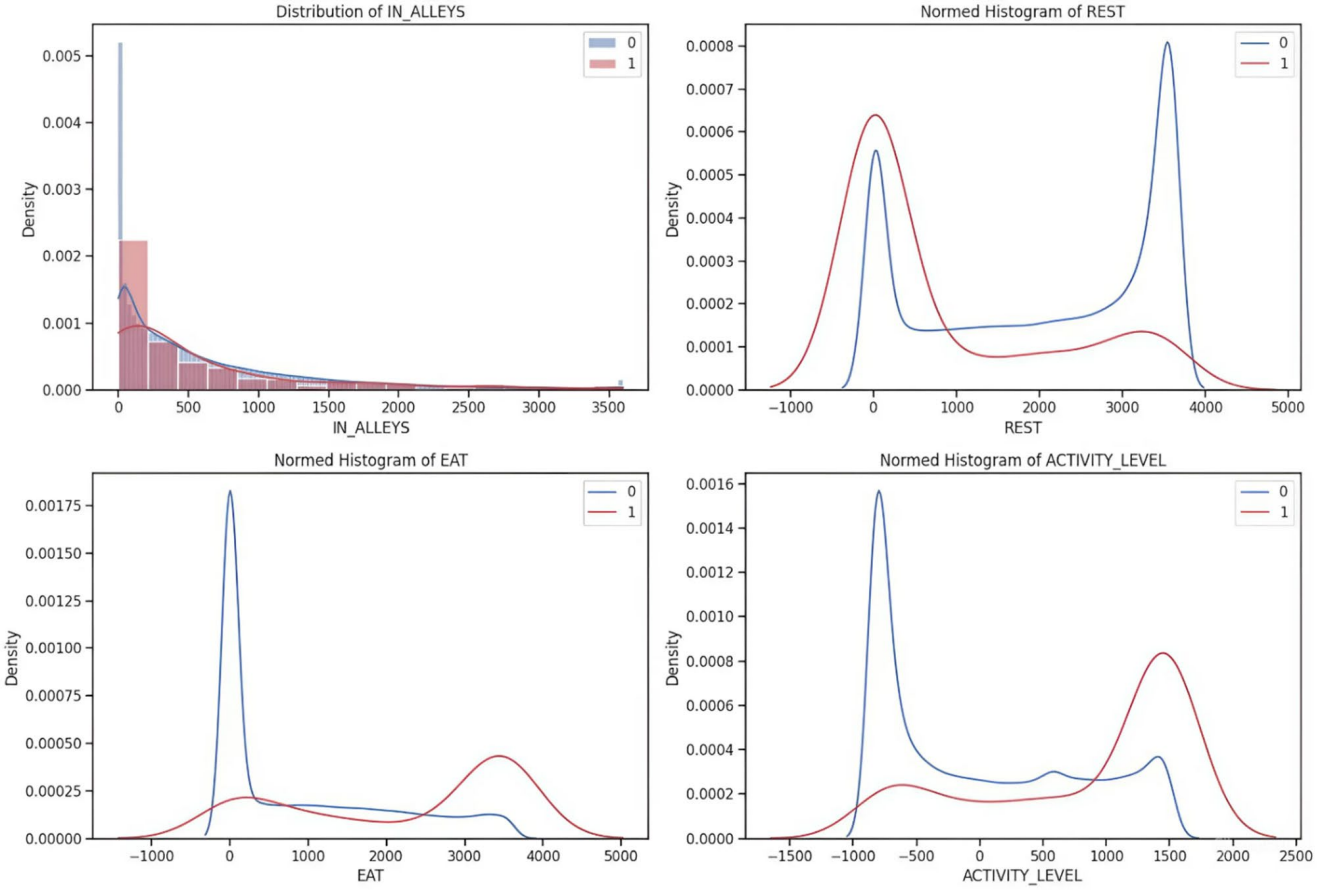
Relationship of behavioral activity concerning Calving. [0(*absence*) & 1(*presence*)]

**Fig. 3:**
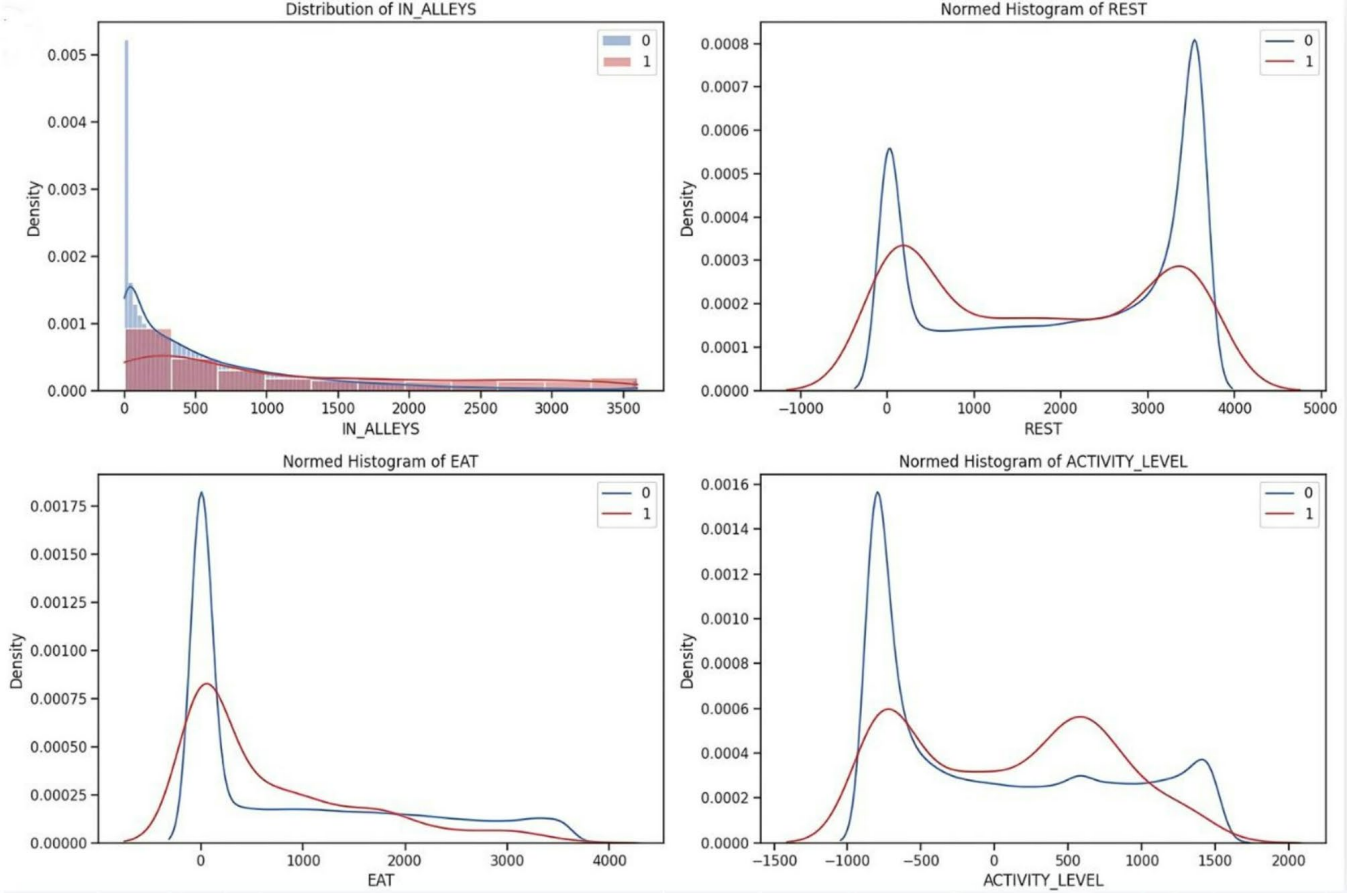
Relationship of behavioral activity concerning Estrus. [0(*absence*) & 1(*presence*)]

**Fig. 4:**
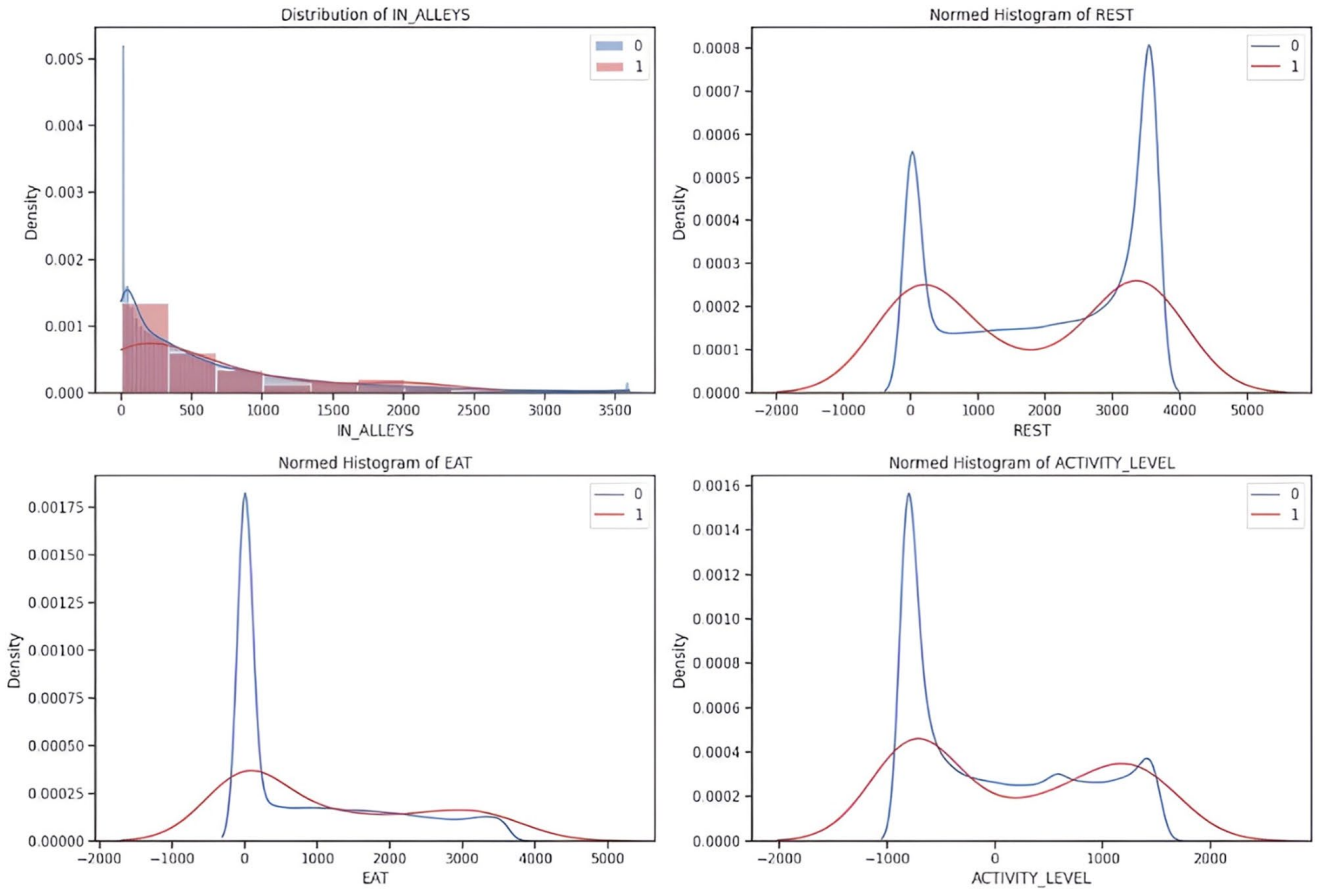
Relationship of behavioral activity concerning Mastitis. [0(*absence*) & 1(*presence*)]

**Fig. 5:**
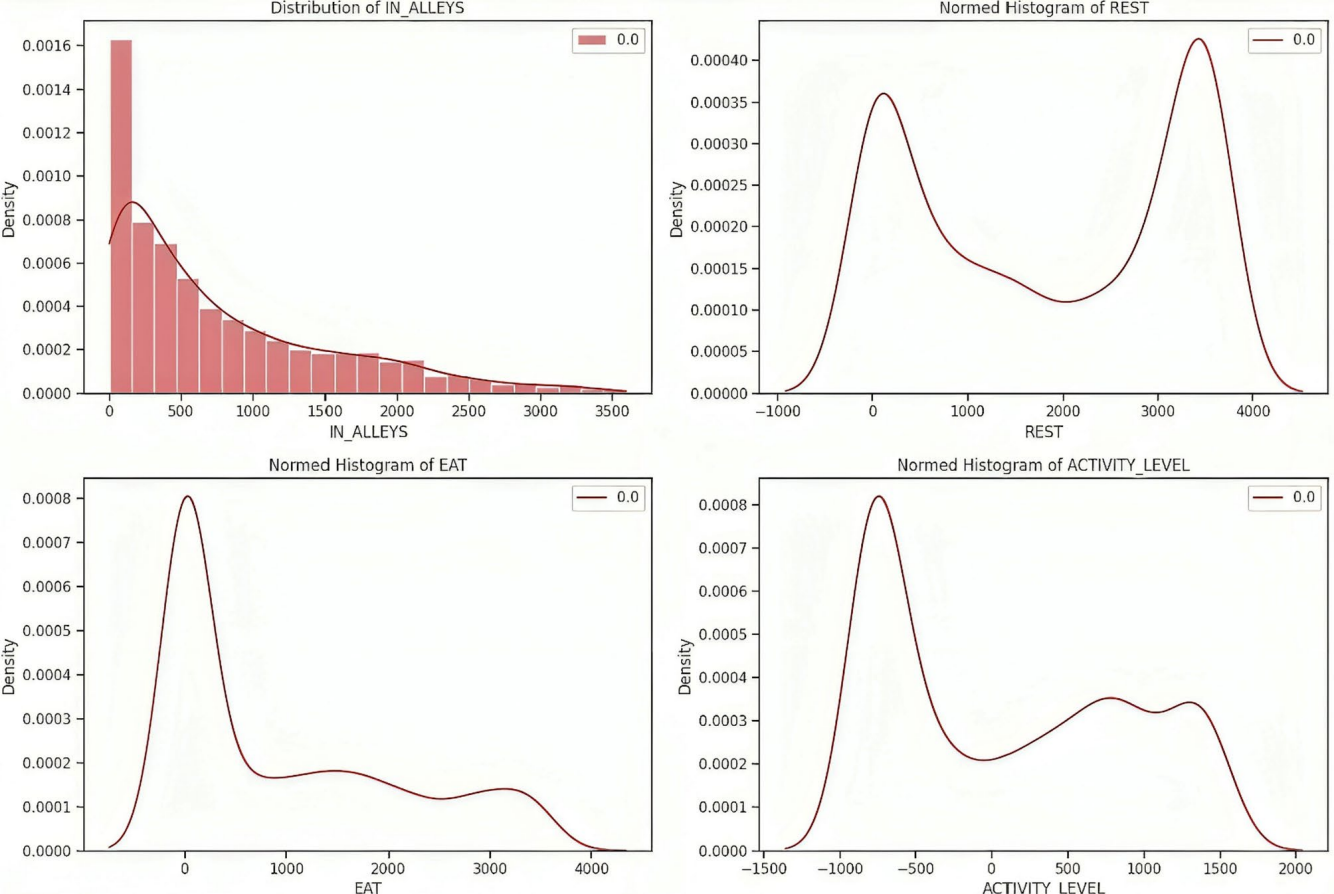
Relationship of behavioral activity concerning Acidosis. [0(*absence*) & 1(*presence*)]

**Fig. 6:**
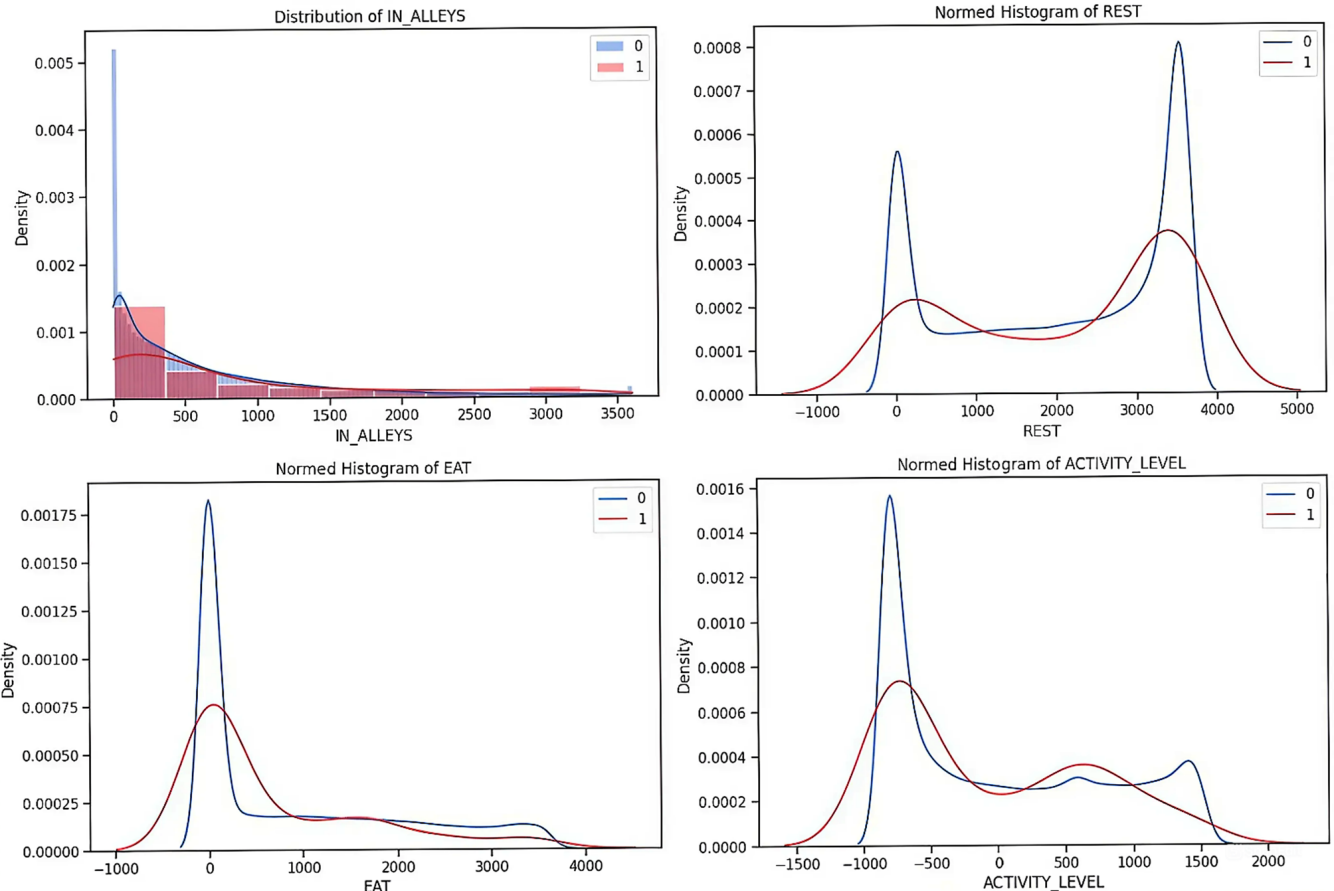
Relationship of behavioral activity concerning Lameness. [0(*absence*) & 1(*presence*)]

The cleaned dataset is subjected to exploratory data analysis to uncover important insights about the relationships between the input features and target labels^20^. EDA includes the density plots that provide a deeper understanding of the dataset and are used to visualize the distributions of input features like EAT, REST, IN-ALLEYS, and ACTIVITY-LEVEL concerning the presence or absence of each target label calving, estrus, mastitis, acidosis, and lameness respectively in the Figs. 2, 3, 4 and 5, and 6. This analysis serves as a precursor to feature selection by highlighting potential relationships between the features and the target labels. However, no feature reduction is performed at this stage to avoid prematurely eliminating useful data.

The distribution plots show the distribution of time spent in alleys, resting, eating, and activity levels under different health conditions. Features like IN-ALLEYS and EAT show significant right-skewed distributions, with most of the data concentrated on the lower range of values. However, the differences between class distributions suggest that certain health conditions are associated with either reduced or increased time in these activities. Similarly, the bimodal distributions observed for REST and ACTIVITY-LEVEL highlight distinct behavioral patterns, where healthy and unhealthy livestock exhibit different activity or rest levels, creating the potential for these features to contribute to accurate predictions. These visual analyses underscore the importance of selecting the most informative features, as some features may provide more discriminative power than others. The overlap in class distributions suggests that feature selection techniques such as ANOVA, Information Gain, RF, and XGB will be essential for ranking features based on their relevance to the target labels. Additionally, the varying class densities indicate potential data imbalance, reinforcing the necessity of employing techniques like SMOTE to ensure balanced model training.

### Data preprocessing

All relevant information from the dataset is first split into three different sets: training 70%, validation 15%, and testing 15%. Following this, since there would be a class imbalance, SMOTE would be used only for the training set. SMOTE generates synthetic samples for underrepresented diseases like acidosis so that the model does not bias with the more frequent conditions like estrus and mastitis.

### Feature selection

The feature selection reduces the dimensionality of the input data by retaining only the most relevant features. The study employs ANOVA, information gain, RF, and XGBoost feature selection techniques. These are applied exclusively to the training data, ensuring that the model learns to generalize based on the selected features without bias from the unseen data. ANOVA identifies IN_ALLEYS as the most influential feature, followed by REST, EAT, and ACTIVITY_LEVEL. Information gain identifies IN_ALLEYS, REST, ACTIVITY_LEVEL, and EAT as the most predictive behavioral features. The RF highlights IN_ALLEYS as the most significant feature, followed closely by ACTIVITY_LEVEL, REST, and EAT. XGBoost highlights EAT as the most critical feature, followed by IN_ALLEYS, REST, and ACTIVITY_LEVEL.

### Classifier chain models

**R1.C4**: To model the multi-label nature of the target output, where bovines can simultaneously suffer from multiple health conditions, we employ CCMs. Classifier chains allow models to handle correlated labels by treating the predictions of one label as inputs to predict the next label^21,22^. **R2.C7**: Six ML algorithms are integrated into classifier chains, including RF, XGBoost, LightGBM, Extra Trees Classifier, Cat-Boost Classifier, and gradient boosting classifier. These models can handle interdependent labels, complex feature relationships, and imbalanced data, making them highly suited for accurate and efficient multi-label classification in bovine disease detection. **R1.C8**: RF and Extra Trees provide robustness and interpretability, which are crucial for understanding the behavioral features influencing bovine health events. RF uses bootstrap aggregation and random feature selection to create stable, high-performing models with clear feature importance insights. Extra Trees, being more randomized, selecting split thresholds at random rather than through optimization, further reduces variance and speeds up computation, making it particularly effective for handling noisy or highly variable behavioral data. XGBoost and LightGBM deliver highly accurate and efficient predictions through advanced gradient boosting with built-in regularization. XGBoost excels at controlling overfitting via L1/L2 penalties and learning rate tuning, effectively capturing complex label dependencies. LightGBM leverages histogram-based binning and leaf-wise tree growth to scale efficiently on large datasets, making it well-suited for modeling detailed behavioral patterns with fine granularity. HistGradientBoostingClassifier enhances training speed through histogram-based optimization while maintaining accuracy, offering high efficiency on large-scale farm datasets. CatBoost, though often optimized for categorical inputs, remains advantageous in this context due to its ordered boosting strategy, which reduces overfitting and prediction shift even when applied to continuous behavioral features. Its fast convergence and low sensitivity to hyperparameter tuning further support stable and consistent multi-label predictions. A summary of each model’s key strengths and its relevance to the behavioral disease detection framework is presented in Table 4, highlighting how their distinct characteristics contribute to the performance and adaptability of the classifier chain-based approach.

**Table 4:** Summary of model strengths and relevance.

Model	Strengths	Relevance to Problem
Random forest (RF)	Robust, interpretable, stable with bootstrap sampling	Understands behavioral feature impact; reliable for baseline comparison
Extra trees	More randomized than RF, lower variance, faster computation	Handles variability in behavioral data; prevents overfitting in noisy environments
XGBoost	High accuracy, regularization (L1/L2), fine-tuned control	Captures label dependencies without overfitting; strong on imbalanced datasets
LightGBM	Fast training, histogram binning, leaf-wise growth	Scalable for large behavioral datasets; efficient multi-label learning
HistGradientBoosting	Histogram-based optimization, fast training, native missing value support	Efficient on real-world, large-scale farm data
CatBoost	Ordered boosting, fast convergence, low tuning sensitivity	Reduces prediction shift and overfitting; stable for complex multi-label interactions

**Fig. 7:**
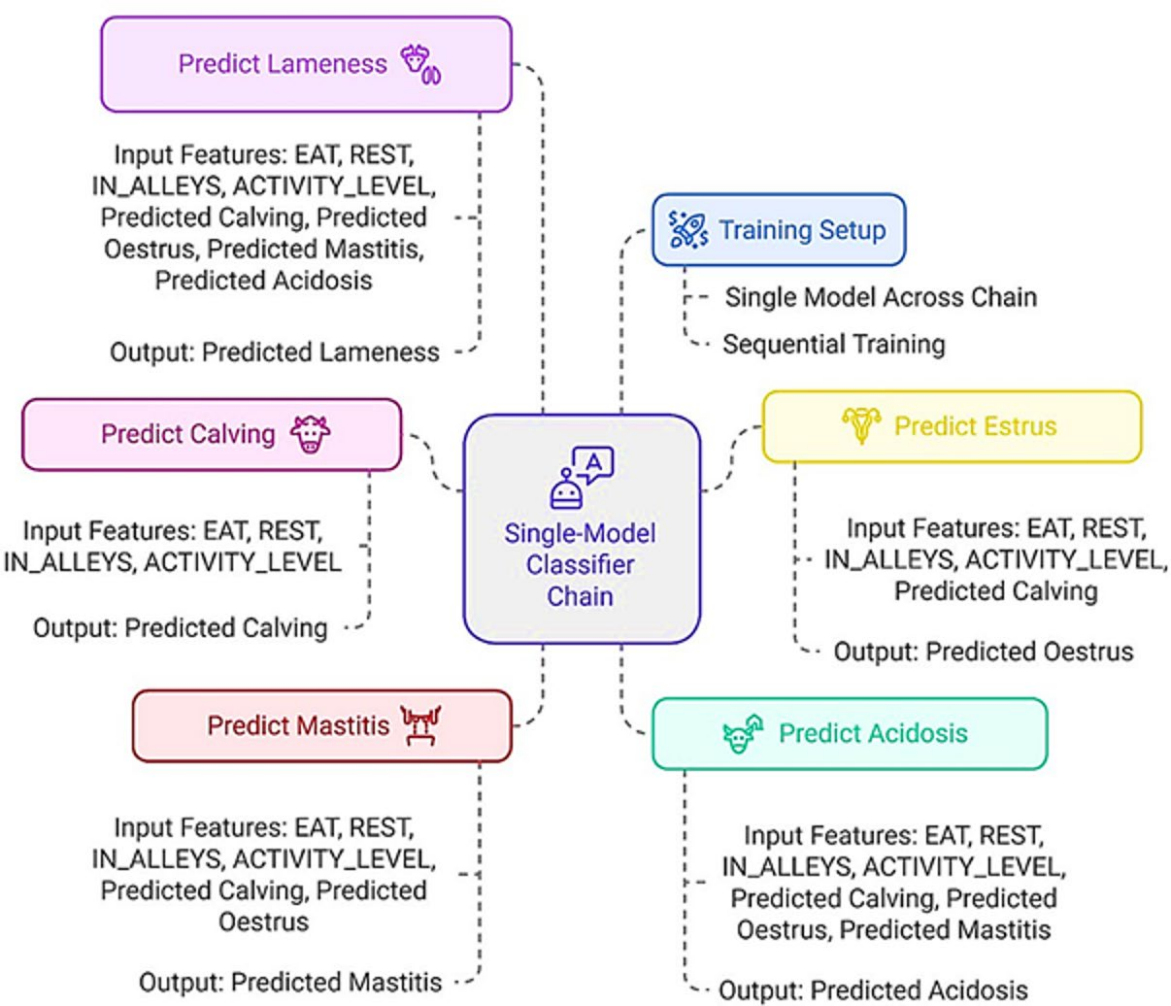
A sequential multi-label classification approach for bovine disease detection, utilizing a single-model classifier chain.

In a single-model classifier chain approach, each model in the sequence is responsible for predicting one specific label, and its output is appended as an additional input feature for the subsequent model in the chain^22^. **R1.C7**: This sequential structure allows the model to exploit interdependencies between labels, enabling more informed and context-aware predictions by incorporating earlier label decisions into subsequent ones. **R1.C9**: Fig. 7 presents a sequential multi-label classification approach for bovine disease detection, utilizing a single-model classifier chain. This architecture ensures that each disease label is predicted in a defined sequence, where the prediction of each previous label is incorporated as an additional input feature for the next. This sequential flow allows the model to capture interdependencies among diseases such as estrus, calving, mastitis, acidosis, and lameness, conditions that often exhibit behavioral overlap. By employing a single underlying base learner across the chain, the framework maintains consistency in learning while preserving the dependencies between labels. The classifier chain structure illustrated in the figure directly supports the proposed methodology’s aim of improving multi-label prediction accuracy by learning not only from the behavioral input features (REST, EAT, IN_ALLEYS, ACTIVITY_LEVEL) but also from the evolving pattern of predicted labels across the chain. The sequential flow, as visualized in Fig. 7, thus forms the core mechanism through which complex label correlations in bovine health monitoring are effectively learned and exploited.

**R1.C10**: The labels in the classifier chain were arranged in a biologically informed sequence: Calving → Estrus → Mastitis → Acidosis → Lameness, reflecting a natural progression of physiological events and disease dependencies in dairy cows. This sequencing enables the classifier chain to exploit meaningful correlations between conditions, rather than treating each disease independently. The rationale for this order lies in the cascading biological impacts following calving. Calving initiates substantial hormonal shifts, behavioral changes, and physiological stress, which directly influence the onset of subsequent conditions. For instance, estrus prediction becomes more relevant after calving, as cows naturally return to estrus if not impregnated. The transition to estrus can affect activity patterns and increase vulnerability to further complications such as mastitis and lameness^23,24^. Mastitis is often observed in newly calved cows, especially under stress or due to poor milking hygiene, making its prediction post-calving or post-estrus biologically coherent^25^. Acidosis, primarily metabolic, tends to emerge following mastitis or dietary changes common in high-yielding cows post-calving or during estrus. It disrupts rumen function and feeding behavior, and as such, logically follows these events in the chain^26^. Finally, lameness is positioned last, as it may arise from the compounded effects of reproductive activity, metabolic imbalances (e.g., acidosis), and extended physical stress, such as prolonged standing or irregular gait during estrus^27^. This biologically grounded label ordering not only enhances predictive accuracy but mirrors real-world disease development pathways in bovine health monitoring systems.

The training is set up sequentially, employing a single model across the entire chain. This classifier chain integrates dependencies among the different health conditions, making the approach more robust than treating each prediction independently. It leverages direct sensor data and previously predicted disease conditions, allowing the system to capture complex interrelations between various bovine health parameters. Utilizing this chain gives the model a richer context, especially when diseases like mastitis or estrus might contribute to the likelihood of other conditions like lameness or acidosis. This design also supports multi-label classification, where each label corresponds to a different health event or disease. The diagram emphasizes how interlinked diseases can be predicted by capitalizing on each preceding model’s output to improve predictive accuracy.

### Validation

After optimal features have been chosen from the training data, those same features should subsequently be used on the validation and test sets. Such similarities will allow the model to be evaluated consistently across all datasets. We created a dedicated validation set for hyperparameter tuning and for preliminary performance assessment of six ML models within the CCM framework, ensuring good performance for the model and counteracting overfitting. The dataset was divided into 70, 15, and 15% for training, validation, and testing, respectively.

The validation set was for fine-tuning the model, and it also rendered an unbiased evaluation of each model’s prediction performance before the final tests. The testing set, on the other hand, provides the final evaluation of how well the model generalizes to new data. **R1.C11**: During validation, key multi-label evaluation metrics, including macro-averaged F1-score, hamming loss, subset accuracy, macro precision, and macro recall, were utilized to rigorously assess model performance in capturing multiple, potentially co-occurring disease states. These metrics are particularly important in multi-label classification tasks with imbalanced label distributions, as they provide a more equitable evaluation across both common and rare disease labels. These validation results guided the selection of optimal hyperparameters, ensuring that the models retained their performance on the independent test set, thus confirming their generalization and applicability for practical disease monitoring in dairy cattle.

### Hyper-Parameter tuning

Hyperparameter tuning is essential for emphasizing the need to capture label interdependencies and reduce overfitting in complex, imbalanced data^28^. GridSearchCV was employed to explore combinations of hyperparameters for each model. These hyperparameters are tailored to improve the model’s predictive individual labels and overall performance in capturing the dependencies among target labels in the classifier chain. **R2.C8**: Each of the six machine learning models employed in the classifier chain was carefully fine-tuned based on the observed behavior of bovine health data. For the Random Forest model, which can be prone to overfitting in high-dimensional settings, tuning key hyperparameters such as n_estimators = 300, max_depth = 20, min_samples_split = 4, max_features = ‘sqrt’, and min_samples_leaf = 2 contributed to increased robustness, interpretability, and stable performance in multi-label contexts. Similarly, for XGBoost, effective generalization was achieved by setting n_estimators = 250, learning_rate = 0.05, max_depth = 6, subsample = 0.8, and colsample_bytree = 0.8, which collectively controlled model complexity and improved resistance to overfitting. In the case of the HGBC, optimal efficiency and generalization were attained by fine-tuning parameters including learning_rate = 0.05, max_iter = 200, max_leaf_nodes = 27, max_depth = 13, and min_samples_leaf = 16, making HGBC particularly suitable for large-scale, multi-label prediction. For LightGBM, refined settings such as num_leaves = 42, learning_rate = 0.03, max_depth = 10, feature_fraction = 0.8, and min_child_samples = 19 enhanced its predictive consistency and robustness across complex bovine disease indicators. The Extra Trees Classifier, known for its randomized splitting strategy, was optimized with n_estimators = 350, max_depth = 23, min_samples_split = 3, min_samples_leaf = 5, and max_features = ‘sqrt’, ensuring a strong balance between generalization and variance control. Lastly, the CatBoost Classifier, with its unique ordered boosting and internal regularization mechanisms, mitigated prediction shift and overfitting, even with minimal hyperparameter tuning. Its final optimized configuration iterations = 300, depth = 8, learning_rate = 0.06, and l2_leaf_reg = 7, demonstrated strong multi-label accuracy and stability, particularly in the complex and interdependent nature of bovine disease detection tasks.

### Model evaluation

Given the complexity and interdependence of health conditions such as calving, estrus, mastitis, acidosis, and lameness, a comprehensive set of metrics is employed, as discussed in Table 5, to assess the efficacy of the proposed bovine disease detection system. These include per-label metrics and overall multi-label metrics to ensure that the model not only predicts each health condition accurately but also respects label dependencies. A multi-label classifier for predicting bovine health conditions is formed and conducted by the presented methodology. To build such a system, stratified data splitting is done, along with the application of SMOTE to cure class imbalance, selection of highly predictive features, and usage of CCMs to depend on the correlation among health events, ensuring accurate predictions. Hyperparameter tuning and evaluation ensure that the models are tuned toward generalization on unseen data. The pipeline also presents and incorporates techniques for feature scaling and normalization to ensure that all features are regularly scaled down and free from bias by a few features.

## Experimentation results and evaluation

This section introduces the performance outcomes of the proposed CCMs in the prediction of various bovine diseases and events with the use of six ML classifiers. The models were on the training set, optimized on the validation set, and evaluated on the test set for their generalizability. ANOVA, Information Gain, RF, and XGBoost feature selection were performed to rank behavioral features REST, EAT, IN_ALLEYS, and ACTIVITY_LEVEL according to their contribution towards target label predictions. While different techniques ranked features differently in terms of importance, all of them demonstrated sufficient importance, leaving no single feature to be of such limited utility that it was dropped. Validation scores were utilized to fine-tune each model’s parameters, ensuring robust performance on unseen data. The evaluation metrics include both per-label and overall multi-label measures. Per-label metrics assess the models’ ability to accurately predict individual health events. Whereas multi-label metrics evaluate the overall model performance in handling interdependencies between health events and imbalanced datasets.

**Table 5:** Comprehensive evaluation metrics framework for per-label and overall metrics for assessing model performance in multi-label bovine disease detection.

Metric Type	Metric Name	Description
Per-label metrics	Accuracy	It measures how often a model’s predictions are correct.
	Precision	Indicates the percentage of correctly identified positive instances out of all predicted positives.
	Recall	Measures the proportion of true positive predictions among all actual positives.
	F1-Score	The harmonic mean of precision and recall provides a balanced measure and indicates potential class imbalance.
Overall multi-label metrics	Macro-averaged precision	Calculates the average precision across all labels, treating each label equally regardless of frequency.
	Macro-averaged recall	Identify true positive cases for each disease label, treating all labels equally regardless of frequency.
	Macro-averaged F1-score	The mean F1-score for all labels, ensuring equal consideration of rare and frequent labels, provides a balanced measure of macro-precision and macro-recall across all disease labels.
	Subset accuracy	Measures the proportion of instances where the model correctly predicts all labels for a given instance.
	Hamming loss	Measures the fraction of misclassified labels, providing insight into the model’s error rate across all disease labels for each instance.

It can be seen from Table 6 that RF demonstrated exceptional performance post-tuning, achieving F1-scores above 98% for most events on the test set, notably excelling in predicting calving and lameness with near-perfect metrics. However, extreme gradient boost showed moderate results across the board, particularly underperforming for acidosis, obtaining an F1-score of 90% despite high precision values for other conditions, outperforming for the event of estrus with the F1-score and precision of 98%. Similarly, CatBoost, known for its versatility, exhibited consistent performance across all labels. It achieved post-tuning F1 scores in the 97% − 98% range for most events, with stable recall for rarer conditions, where it performed best for the calving observations.

Furthermore, it is evident through results that Hist Gradient Boost, while competitive for calving and estrus, struggled with acidosis during validation, getting the F1-score of 61% but improved to 90% post-tuning on the test set. Notably excels in predicting lameness with near-perfect metrics. Compared to all, the extra trees classifier emerged as a standout performer, maintaining near-perfect F1-scores and other metrics for all events after tuning, effectively managing imbalanced datasets. However, light gradient boosting achieved balanced precision and recall across all events, particularly excelling in calving and estrus, with F1-scores around 97% post-tuning.

The per-label evaluation emphasizes the critical impact of hyperparameter optimization in refining classifier performance, mainly for interdependent conditions. Models like extra trees and light gradient boost consistently demonstrated excellence, as reflected in their post-tuning scores, which surpassed 95% for most events. By leveraging interdependencies, the CCM effectively captured correlations between diseases, such as lameness and mastitis. This capability is critical for improving predictions in complex, real-world datasets^29^. Besides, multi-label measures were used to evaluate the overall efficacy of the CCM. Table 7 provides a consolidated view of multi-label metrics across classifiers, indicating how well each model handles frequent and rare diseases across different metrics.

**Table 6:** Performance metrics of the overall classification on per-label metrics for each model on the validation and test sets after hyperparameter tuning.

Model	Event	Validation set				Test set			
		Accuracy	Precision	Recall	F1-Score	Accuracy	Precision	Recall	F1-Score
Random Forest	Calving	86%	82%	93%	87%	98%	97%	99%	98%
	Estrus	85%	80%	91%	85%	97%	95%	96%	98%
	Mastitis	80%	77%	85%	81%	97%	94%	87%	89%
	Acidosis	77%	74%	82%	78%	95%	92%	94%	94%
	Lameness	82%	79%	87%	83%	97%	96%	96%	98%
Extreme Gradient	Calving	77%	74%	85%	80%	97%	99%	97%	95%
	Estrus	79%	74%	84%	80%	98%	98%	97%	98%
	Mastitis	80%	76%	81%	82%	96%	96%	98%	97%
	Acidosis	76%	73%	79%	77%	90%	86%	94%	90%
	Lameness	75%	72%	80%	76%	95%	96%	97%	97%
Cat-Boost	Calving	79%	77%	86%	81%	98%	98%	98%	98%
	Estrus	77%	78%	81%	79%	97%	96%	99%	97%
	Mastitis	76%	72%	86%	78%	97%	97%	98%	97%
	Acidosis	74%	70%	79%	72%	94%	92%	98%	95%
	Lameness	75%	78%	84%	82%	88%	86%	92%	90
Hist-Gradient	Calving	73%	76%	66%	71%	94%	92%	98%	95%
	Estrus	76%	77%	80%	76%	96%	90%	90%	94%
	Mastitis	79%	75%	80%	81%	95%	90%	94%	92%
	Acidosis	65%	67%	57%	61%	89%	87%	88%	90%
	Lameness	76%	72%	85%	75%	95%	89%	98%	97%
Extra Tree	Calving	87	83%	92%	87%	99%	99%	100%	100%
	Estrus	85%	82%	90%	86%	99%	98%	100%	99%
	Mastitis	85%	80%	87%	90%	97%	98%	98%	97%
	Acidosis	79%	74%	80%	81%	95%	97%	96%	94%
	Lameness	84%	79%	86%	84%	96%	99%	100%	99%
Light Gradient	Calving	83%	80%	91%	85%	98%	99%	98%	96%
	Estrus	85%	82%	91%	87%	97%	98%	97%	97%
	Mastitis	79%	81%	80%	82%	95%	90%	93%	96%
	Acidosis	73%	76%	66%	71%	91%	89%	90%	90%
	Lameness	74%	71%	83%	76%	95%	97%	94%	96%

It can be seen from Table 7 that the extra trees classifier achieved a subset accuracy of 97% and a macro-averaged F1 score of 96%, showcasing its effectiveness in predicting both frequent and rare events with minimal misclassification. Similarly, light gradient boosting followed closely, with a macro-averaged F1 score of 95% and hamming loss of 0.04, highlighting its reliability for imbalanced datasets. On the contrary, despite improvement post-tuning, the hist gradient boosting lagged in subset accuracy due to challenges in predicting rarer events like acidosis. Models incorporating SMOTE-based pre-processing techniques showcased notable improvements in handling imbalanced data distributions, effectively capturing correlations between co-occurring conditions such as mastitis and lameness.

The thorough analysis in Table 7 reinforces the CCM’s ability to transform bovine health diagnostics by providing dependable, real-time predictions of interconnected health events. Extra trees consistently outperformed the other classifiers, achieving test F1 scores of over 90% for all events. Light gradient boost and random forest closely followed, showcasing their strength in generalization and accuracy. On the other hand, the hist-gradient boost faced challenges at first but showed significant improvement after adjustments. While Cat-Boost maintained stability, its performance was slightly lower than that of Extra Trees. The extreme gradient boost demonstrated potential but was inconsistent in some rare conditions. **R2.C2**: These differences underscore the significance of selecting the right model and fine-tuning hyperparameters to enhance multi-label classification.

**Table 7:** Performance metrics of the overall multi-label classification for each model on the validation and test sets.

Model	Dataset	Hamming Loss	Subset Accuracy	Macro Precision	Macro Recall	Macro F1 Score
Random forest	Train	0.05	92%	92%	95%	95%
	Validation	0.06	92%	93%	94%	94%
	Test	0.07	91%	92%	93%	93%
Extra tree	Train	0.05	98%	97%	97%	97%
	Validation	0.05	94%	94%	95%	94%
	Test	0.04	97%	95%	96%	96%
CatBoost	Train	0.05	95%	96%	96%	97%
	Validation	0.06	93%	94%	95%	95%
	Test	0.06	92%	93%	94%	94%
LightGBM	Train	0.04	96%	96%	97%	97%
	Validation	0.05	94%	95%	96%	96%
	Test	0.05	94%	94%	95%	95%
LightGBM	Train	0.04	94%	94%	96%	96%
	Validation	0.06	92%	93%	94%	94%
	Test	0.07	91%	92%	93%	93%
XGBoost	Train	0.05	95%	96%	96%	97%
	Validation	0.06	93%	94%	95%	95%
	Test	0.06	92%	93%	94%	94%

The findings indicate that certain models have highly increased early health problem detection in cattle, resulting in better animal welfare, lower economic losses, and increased productivity at the farm. The study reaffirms the critical need to confront imbalanced data and feature selection, thus ensuring the detection system’s reliability and accuracy. This research provides insights that structure the conception of a framework that uses machine learning to deal with detection problems, with a particular emphasis on how best to employ techniques like SMOTE to increase detection rates for rare diseases such as acidosis. Most importantly, the diseased field studies were capable of recognizing everyday illnesses, including Estrus and Mastitis, boasting high F1 scores. It has also effectively allowed capturing the interdependencies of the highly concomitant conditions of lameness and mastitis. To ensure the generalizability of the proposed framework, a stratified 10-fold cross-validation method was used to make sure that the proposed machine learning framework was reliable and could be used in a wide range of situations. **R2.C2**: This method trains and tests the model on different parts of the dataset, which helps to reduce the risk of performance inflation caused by data-specific biases. Table 8 shows a comparison of the results from the training and test sets for all the models that were looked at across five bovine health conditions: calving, estrus, mastitis, acidosis, and lameness. The accuracy, precision, recall, and F1-score of the reported test set performance metrics, averaged across the cross-validation folds, are very similar to those of the training set. This consistency across evaluation sets shows that the models are still good at predicting things on data they haven’t seen before and are not showing any signs of overfitting. In this case, the Extra Trees classifier got an F1-score of 100% on both the training and test sets for calving, and it got similarly high scores on all other conditions with very few changes. These results show that the model can find meaningful patterns in behavior without overfitting to the training data. This means that it is strong enough to be used in real-world dairy health monitoring systems.

**R2.C3**:

**Table 8:** Performance metrics of the overall classification on per-label metrics for each model to demonstrate overfitting mitigation.

Model	Event	Train set				Test set			
		Accuracy	Precision	Recall	F1-Score	Accuracy	Precision	Recall	F1-Score
Random Forest	Calving	99%	98%	99%	98%	98%	97%	99%	98%
	Estrus	98%	97%	98%	99%	97%	95%	96%	98%
	Mastitis	99%	97%	89%	92%	97%	94%	87%	89%
	Acidosis	97%	95%	98%	95%	95%	92%	94%	94%
	Lameness	98%	98%	98%	99%	97%	96%	96%	98%
Extreme Gradient	Calving	98%	99%	99%	97%	97%	99%	97%	95%
	Estrus	98%	99%	99%	98%	98%	98%	97%	98%
	Mastitis	97%	98%	98%	99%	96%	96%	98%	97%
	Acidosis	94%	90%	97%	94%	90%	86%	94%	90%
	Lameness	97%	98%	98%	99%	95%	96%	97%	97%
Cat-Boost	Calving	98%	99%	100%	99%	98%	98%	98%	98%
	Estrus	99%	98%	99%	99%	97%	96%	99%	97%
	Mastitis	98%	99%	98%	97%	97%	97%	98%	97%
	Acidosis	98%	96%	98%	97%	94%	92%	98%	95%
	Lameness	92%	89%	96%	95%	88%	86%	92%	90%
Hist-Gradient	Calving	97%	97%	99%	98%	94%	92%	98%	95%
	Estrus	98%	95%	96%	95%	96%	90%	90%	94%
	Mastitis	97%	95%	97%	96%	95%	90%	94%	92%
	Acidosis	93%	93%	91%	95%	89%	87%	88%	90%
	Lameness	98%	92%	98%	99%	95%	89%	98%	97%
Extra Tree	Calving	100%	99%	100%	100%	99%	99%	100%	100%
	Estrus	99%	99%	100%	100%	99%	98%	100%	99%
	Mastitis	99%	98%	99%	100%	97%	98%	98%	97%
	Acidosis	99%	99%	99%	98%	95%	97%	96%	94%
	Lameness	99%	100%	100%	100%	96%	99%	100%	99%
Light Gradient	Calving	99%	100%	98%	98%	98%	99%	98%	96%
	Estrus	98%	98%	99%	99%	97%	98%	97%	97%
	Mastitis	98%	95%	95%	94%	95%	90%	93%	96%
	Acidosis	94%	92%	94%	94%	91%	89%	90%	90%
	Lameness	98%	99%	98%	99%	95%	97%	94%	96%

**R2.C1**: Notably, though the results create strong groundwork for advancement in bovine health monitoring systems, a few important limitations need to be addressed. One major limitation relates to the exclusive usage of behavioral data for infection prediction. Though highly informative, the eating, resting, and activity patterns may be incapable of totally underlie physiological conditions or other environmental factors impacting the health of the cow. Additional data sources, such as biomarkers, genetic information, or environmental conditions (like temperature and humidity), may enhance the accuracy of prediction and thus widen the applicability benefits of the model. Another limitation is generalizability, in that the data set used in the current study was taken from four commercial farms with Holstein cattle, which do not represent wide variations that one might expect from changes in breed, farming practices, or geography^10^.

**R2.C1**: XAI will be very important in the future for making real-world dairy systems better at predicting things. It can prove that the model is flexible and make it easier to use around the world by testing it on different types of cows and in different farming settings. Combining different types of data, like physiological signals and past behaviour patterns, could make predictions more accurate. Advanced deep learning architectures, like recurrent neural networks, could help the model learn more about the complex time-based relationships that happen when a disease starts. Also, adding explainable AI tools like SHAP or LIME could give veterinarians and farmers clear, easy-to-understand reasons for predicted events, which would boost decision confidence and promote openness.

## Conclusion

This work proposed a comprehensive and intelligent multi-label classification framework to predict critical bovine diseases and events with the support of behavioral data, enhancing proactive health management in dairy farming. The design implements a single-shot classifier chain in a broader and proper ML pipeline for data pre-processing, feature selection, SMOTE oversampling, and hyperparameter tuning across the most advanced models. The proposed method effectively captures the intricate interdependencies among several disease labels such as estrus, calving, lameness, mastitis, and acidosis. The multi-label classification framework thus demonstrates how a classifier chain can further extrapolate the predictive capabilities for timely, accurate, and proactive predictions concerning bovine diseases. Such an approach harnessing the combined potential of balanced sampling, interdependent label modeling, and optimal classifiers seeks to address the challenges facing disease detection while availing real-time monitoring, prediction, and response to health risks on farms. This work may provide a conduit for AI-led solutions for enhanced animal welfare and agricultural operational efficiency.

## Data Availability

The dataset used for this study is of dairy cows in barns collected from the CowView system (GEA Farm Technology, Bonn, Germany); refer to Ref. [10]. Additional information is available from the corresponding author upon reasonable request. (Following links are used for the data resources.).https://doi.org/10.15454/1.5572318050509348E12https://data.inrae.fr/privateurl.xhtml? token=7e2f5c12-400b-45c4-b543-b512688da799.
